# Chronic kidney disease, atherosclerotic plaque characteristics on carotid magnetic resonance imaging, and cardiovascular outcomes

**DOI:** 10.1186/s12882-021-02260-x

**Published:** 2021-02-24

**Authors:** Srinivasan Beddhu, Robert E. Boucher, Jie Sun, Niranjan Balu, Michel Chonchol, Sankar Navaneethan, Glenn M. Chertow, Raymond Townsend, William Haley, Alfred K. Cheung, Molly B. Conroy, Dominic S. Raj, Dongxiang Xu, Thomas George, Reem Yunis, Guo Wei, Gador Canton, Jeffrey Bates, Jing Chen, Vasilios Papademetriou, Henry Punzi, Alan Wiggers, Jackson T. Wright, Tom Greene, Chun Yuan

**Affiliations:** 1 Medical Service, Veterans Affairs Salt Lake City Health Care System, Salt Lake City, USA; 2 Division of Nephrology & Hypertension, University of Utah School of Medicine, 85 North Medical Drive East, Room 201, Salt Lake City, UT 84112 USA; 3 Department of Radiology, Vascular Imaging Lab, University of Washington, Seattle, WA USA; 4 Division of Renal Diseases and Hypertension, University of Colorado Anschutz Medical Campus, Aurora, CO USA; 5 Section of Nephrology, Baylor College of Medicine, Houston, TX USA; 6 Section of Nephrology, Michael E. DeBakey Veterans Affairs Medical Center, Houston, TX USA; 7 Division of Nephrology, Stanford University School of Medicine, Palo Alto, CA USA; 8 Division of Nephrology, University of Pennsylvania, Philadelphia, PA USA; 9 Division of Nephrology, Mayo Clinic, Jacksonville, FL USA; 10 Division of General Internal Medicine, University of Utah School of Medicine, Salt Lake City, UT USA; 11 Division of Nephrology, George Washington University, Washington, DC USA; 12 Division of Nephrology, Cleveland Clinic, Cleveland, OH USA; 13 Medical Care Line, Michael E. DeBakey VA Medical Center, Houston, TX USA; 14 Department of Medicine, Baylor College of Medicine, Houston, TX USA; 15 Tulane University School of Medicine, New Orleans, LA USA; 16 Veterans Affairs Medical Center, Washington, DC USA; 17 Department of Medicine & Clinical Research, Punzi Medical Center, Carrollton, TX USA; 18 Division of Nephrology and Hypertension, Case Western Reserve University, Cleveland, OH USA; 19 Division of Biostatistics, University of Utah School of Medicine, Salt Lake City, UT USA

**Keywords:** Chronic kidney disease, Atherosclerosis, Carotid plaque

## Abstract

**Background:**

It is unclear whether faster progression of atherosclerosis explains the higher risk of cardiovascular events in CKD. The objectives of this study were to 1. Characterize the associations of CKD with presence and morphology of atherosclerotic plaques on carotid magnetic resonance imaging (MRI) and 2. Examine the associations of baseline CKD and carotid atherosclerotic plaques with subsequent cardiovascular events.

**Methods:**

In a subgroup (*N* = 465) of Systolic Blood Pressure Intervention Trial.

(SPRINT) participants, we measured carotid plaque presence and morphology at baseline and after 30-months with MRI. We examined the associations of CKD (baseline eGFR < 60 ml/min/1.73m^2^) with progression of carotid plaques and the SPRINT cardiovascular endpoint.

**Results:**

One hundred and ninety six (42%) participants had CKD. Baseline eGFR in the non-CKD and CKD subgroups were 77 ± 14 and 49 ± 8 ml/min/1.73 m^2^, respectively. Lipid rich necrotic-core plaque was present in 137 (29.5%) participants. In 323 participants with both baseline and follow-up MRI measurements of maximum wall thickness, CKD was not associated with progression of maximum wall thickness (OR 0.62, 95% CI 0.36 to 1.07, *p* = 0.082). In 96 participants with necrotic core plaque at baseline and with a valid follow-up MRI, CKD was associated with lower odds of progression of necrotic core plaque (OR 0.41, 95% CI 0.17 to 0.95, *p* = 0.039). There were 28 cardiovascular events over 1764 person-years of follow-up. In separate Cox models, necrotic core plaque (HR 2.59, 95% CI 1.15 to 5.85) but not plaque defined by maximum wall thickness or presence of a plaque component (HR 1.79, 95% CI 0.73 to 4.43) was associated with cardiovascular events. Independent of necrotic core plaque, CKD (HR 3.35, 95% CI 1.40 to 7.99) was associated with cardiovascular events.

**Conclusions:**

Presence of necrotic core in carotid plaque rather than the presence of plaque per se was associated with increased risk of cardiovascular events. We did not find CKD to be associated with faster progression of necrotic core plaques, although both were independently associated with cardiovascular events. Thus, CKD may contribute to cardiovascular disease principally via mechanisms other than atherosclerosis such as arterial media calcification or stiffening.

**Trial Registration:**

NCT01475747, registered on November 21, 2011.

**Supplementary Information:**

The online version contains supplementary material available at 10.1186/s12882-021-02260-x.

## Background

There is a substantially higher incidence of cardiovascular (CV) events in the chronic kidney disease (CKD) population compared to the general population [1–4]. Nearly 45 years ago, Lindner et al. proposed the paradigm of accelerated atherosclerosis in uremia [5]. However, it remains unclear the extent to which atherosclerotic and non-atherosclerotic processes contribute to the CV events in CKD. Some studies suggest greater prevalence of atherosclerotic burden as assessed by carotid and/or femoral ultrasound, particularly in persons on chronic maintenance hemodialysis [6, 7] and to a lesser extent in non-dialysis dependent CKD [8–10]. However, some longitudinal studies have not shown faster progression of atheroma defined by intima-media thickness measured with ultrasound in persons with CKD [11–13]. This issue raises the possibility that CKD might not be associated with progression of intima-media thickness on ultrasound but still associated with altered plaque characteristics predictive of increased CV risk.

High-resolution magnetic resonance imaging (MRI) techniques have made direct assessment of plaque tissue composition possible. Carotid atherosclerosis as quantified by MRI scans showed excellent correlation with carotid histology performed in ex vivo human carotid endarterectomy specimens [14] . Furthermore, modified American Heart Association (AHA) classification of carotid plaques with in vivo MRI scans performed prior to carotid endarterectomy demonstrated excellent correlation with ex vivo histology of resected carotid specimens [15]. Thus, multi-contrast (which does not require gadolinium contrast) carotid MRI imaging can be used to accurately measure carotid plaque burden [16, 17] and composition [15], including intraplaque hemorrhage [14, 18], fibrous cap [19–21] and lipid-rich necrotic core [14, 22].

The Systolic Blood Pressure Intervention Trial (SPRINT) was a randomized controlled trial that tested the effects of intensive systolic blood pressure (SBP) lowering to a goal of less than 120 mmHg versus a SBP goal of less than 140 mmHg on cardiovascular outcomes [23]. We conducted Systolic Blood Pressure Intervention Trial- Factors affecting Atherosclerosis Study (SPRINT-FAST) as an ancillary study (Clinical Trials registration: NCT01475747) to SPRINT and performed carotid MRIs to characterize the presence and morphology of atherosclerotic plaques in 501 SPRINT participants at 8 SPRINT clinical sites across the US.

Herein, we examined the baseline and longitudinal associations of CKD with the presence and morphology of carotid atherosclerotic plaques, the longitudinal associations of baseline CKD and carotid atherosclerotic plaques with SPRINT CV composite outcome, and whether the presence of CKD at baseline modified the associations of carotid atherosclerotic plaques with the SPRINT CV composite outcome.

## Methods

Details of SPRINT inclusion and exclusion criteria and study procedures have been published elsewhere [23, 24]. All SPRINT participants within eighteen months of randomization at eight SPRINT sites were potentially eligible to participate in SPRINT FAST. Those who gave informed consent and did not have contraindications to MRI were included in SPRINT FAST.

### Definition of CKD

SPRINT used the 4-variable Modification of Diet in Renal Diseases (MDRD) equation. SPRINT CKD subgroup was pre-specified in the SPRINT protocol as those with as baseline eGFR < 60 ml/min/1.73 m^2^ at SPRINT randomization. Any SPRINT participant defined to have CKD at SPRINT baseline was considered to have CKD for SPRINT-FAST.

### Carotid MRI protocol and analyses

Participants were scanned at SPRINT-FAST baseline with a follow-up scan at 30 months on 3 T MRI scanners using a multi-contrast carotid protocol developed by the SPRINT-FAST MRI Reading Center at the University of Washington Vascular Imaging Laboratory. This standardized protocol included stringent quality control measures [25, 26] (details in supplemental methods). In brief, the protocol included two-dimensional T1-weighted (T1w), T2-weighted (T2w), and proton-density-weighted (PDw) fast spin echo sequences with double inversion-recovery preparation as well as three-dimensional time-of-flight (TOF) and magnetization-prepared rapid acquisition gradient echo (MP-RAGE). All images were acquired in the axial plane with 0.63mmx0.63 mm in-plane resolution and 3 mm (1 mm for TOF) cross-sectional slices, centered on the flow divider of the extracranial carotid artery bifurcation.

**Fig. 1:**
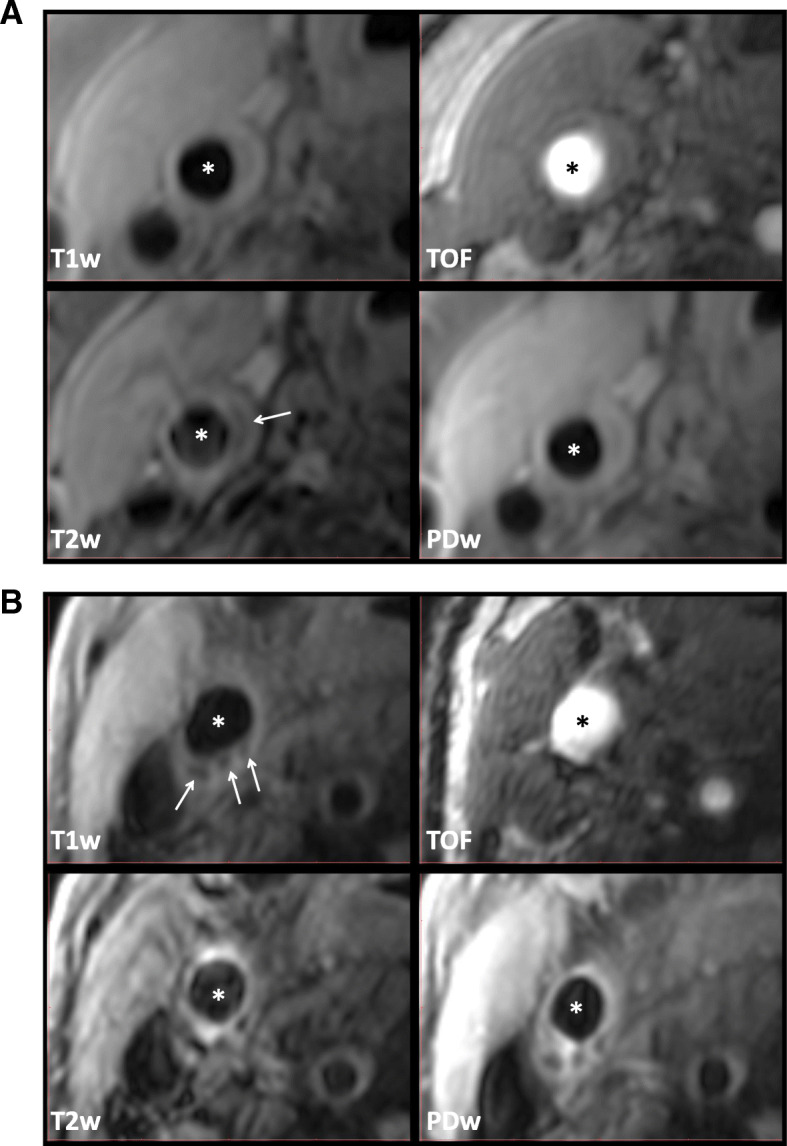
Magnetic resonance (T1 weighted, time of flight, T2 weighted and proton-density-weighted) images of carotid plaques with a necrotic core (panel **a**) and calcification (panel **b**). * represents lumen and arrows point to lesions in common carotid artery before carotid bifurcation

Carotid MR image analyses were performed centrally at the SPRINT-FAST MRI Reading Center with an in-house software (CASCADE, Vascular Imaging Lab, Seattle, WA). Inner and outer boundaries of carotid artery wall were traced to measure wall area and wall thickness. Based on previously published criteria [14, 17, 25], plaque components including lipid-rich necrotic core, calcification and intra-plaque hemorrhage were characterized (Fig. 1).

To assess carotid plaque progression or regression during the 30-month study period, the baseline and follow-up scans were matched, again using the carotid bifurcation as the landmark. The right or left carotid artery segment that was covered by scans at both time points was used to measure changes in carotid wall area, lipid-rich necrotic core, and calcification. The trained MRI analysts were blinded to whether the images were baseline or follow-up studies, CKD status, SPRINT intervention status and other clinical data.

#### Definition of carotid atheromatous plaque

The presence of any carotid plaque was defined as maximum wall thickness (MWT) > 1.5 mm in any of the slices or plaque components (lipid rich necrotic core, calcification or intra-plaque hemorrhage). Based on the plaque morphology, plaques with a lipid-necrotic core (NC+ plaques) or calcification (Ca + plaques) were defined. As there were only 14 (3.0%) plaques that had intra-plaque hemorrhage and all the plaques with intra-plaque hemorrhage had a necrotic core, these were included as NC+ plaques in subsequent analyses.

#### Definition of CV outcome

The primary endpoint in SPRINT was a composite of non-fatal myocardial infarction, acute coronary syndrome not resulting in myocardial infarction, stroke, acute decompensated heart failure, or death from CV causes adjudicated by the SPRINT Morbidity and Mortality Committee.

### Statistical methods

#### Baseline associations

We performed all analyses in STATA version MP 15.1, and used a 2-sided α = 0.05 for hypothesis testing, without adjustment for multiple comparisons. We compared baseline characteristics between CKD and non-CKD groups using 1-way analysis of variance (ANOVA) for numeric variables (after log transformation for urine albumin creatinine ratio) and used chi-square tests for categorical variables. We compared baseline MRI characteristics similarly using chi-square tests and Wilcoxon rank-sum tests. We analyzed the association of CKD with presence or absence of baseline plaque types using separate logistic regression models relating CKD status with maximum wall thickness > 1.5 mm, NC+ plaque, and CA+ plaque, adjusting for age, gender, race, CVD history, statin use, and Framingham risk score (which incorporates serum total and HDL cholesterol levels in risk calculation) in all 465 participants.

#### Longitudinal analyses of maximum wall thickness

Comparison of the change in CKD and non-CKD subgroups was the pre-specified primary analyses for this SPRINT ancillary study. In those with baseline and follow-up MRIs with valid maximum wall thickness measurements (*N* = 323), we related baseline CKD status to <− 20, − 20% to 20, > 20% longitudinal change in maximum wall thickness in an ordinal logistic regression model adjusting for age, gender, race, CVD history, Framingham risk score, statin use and BP intervention arm.

#### Longitudinal analyses of plaque presence and plaque morphology

Longitudinal analyses of change in necrotic core volume and calcification volume were challenged by the large number of participants with zero values for the change as shown by the histograms in Supplemental Fig. 1. Furthermore, only four of the participants without NC+ plaques at baseline developed new NC+ plaques and none of the participants without Ca + plaques at baseline developed new Ca + plaque. Therefore, longitudinal analyses of change were limited to those with follow-up MRI and baseline NC+ plaque (*N* = 96) or Ca + plaque (*N* = 147). Longitudinal analyses were performed to relate baseline CKD status with longitudinal changes in NC+ plaque volume, and Ca + plaque volume with models as described above for maximum wall thickness.

In sensitivity analyses, we used spline regression models to relate baseline eGFR as a continuous variable with baseline presence and longitudinal progression of plaques.

#### Associations with CV outcome

We analyzed the association of baseline plaque types and CKD with the SPRINT CV outcome by using separate Cox proportional hazard regression models, for each of any plaque, NC+ plaque, and Ca + plaque, in addition to the CKD subgroup as binary predictors, and covariate adjustment for age, gender, race, CVD history, Framingham risk score group, statin use, and BP intervention arm and stratified by MRI site. Proportionality assumptions were tested and were not violated.

## Results

The current analysis included 465 SPRINT participants who had a SPRINT-FAST baseline carotid MRI that met quality control (Supplemental Fig. S2). Mean baseline age was 71 ± 8 years, 40% were women and 14% were African-American. The median difference between SPRINT baseline visit and SPRINT-FAST baseline MRI was 11.5 (interquartile range 7.8 to 17.9) months.

There were 196 (42%) participants with CKD at baseline. The baseline characteristics in non-CKD and CKD subgroups are summarized in Table 1. The mean baseline eGFR in the non-CKD and CKD participants were 77 ± 14 and 49 ± 8 ml/min/1.73 m^2^, respectively. Participants with CKD were older, more likely to be women and had higher pulse pressure and albuminuria. Baseline characteristics of the CKD subgroups in the current SPRINT ancillary study and parent SPRINT cohort are summarized in Supplemental Table 1. While baseline eGFR, age, systolic and diastolic blood pressures and albuminuria were similar in the two groups, the current study participants were more likely to be women, less likely to be of black race, smoker and had lower prevalence of baseline cardiovascular disease.

**Table 1:** Baseline clinical characteristics by CKD^c^ subgroups

	Non-CKD^c^ Subgroup (*N* = 269)	CKD^c^ Subgroup (*N* = 196)	*P*-value
Estimated GFR^d^, (ml/min/1.73 m2)	77 ± 14	49 ± 8	
Age, (year)	70 ± 8	73 ± 8	< 0.001
Age ≥ 75, (%)	30	42	0.011
Female sex, (%)	33	50	< 0.001
Black race, (%)	13	16	0.46
Aspirin use, (%)	52	55	0.56
Statin use, (%)	42	46	0.39
Past or current smoker, (%)	49	44	0.32
SPRINT intensive SBP arm, (%)	55	51	0.44
Systolic blood pressure, (mmHg)	139 ± 14	141 ± 14	0.075
Diastolic blood pressure, (mmHg)	78 ± 10	76 ± 11	0.082
Pulse pressure, (mmHg)	61 ± 13	65 ± 13	0.001
CVD subgroup^a^, (%)	9	13	0.23
High Framingham risk subgroup^b^, (%)	89	85	0.15
Urine albumin creatinine ratio, (mg/g)	8 (5, 14)	11 (6, 41)	< 0.001
Fasting LDL cholesterol (mg/dl)	110 ± 30	110 ± 32	0.96
Fasting HDL cholesterol (mg/dl)	55 ± 15	54 ± 15	0.52

Results are presented as proportions (for binary variables) or as mean ± SD (for continuous variables other than ACR) or as median with interquartile range (for ACR)

^a^CVD subgroup was defined as one or more of MI, ACS, coronary revascularization, carotid revascularization, PAD with revascularization, > 50% stenosis of coronary/carotid/lower extremity artery; or AAA ≥5 mm

^b^Framingham risk score ≥ 15% for 10 years at SPRINT screening

^c^eGFR < 60 at SPRINT screening

^d^Estimated by 4-variable MDRD equation

### Baseline associations of CKD with carotid plaque and plaque morphology

In the entire SPRINT-FAST cohort at baseline, compared to non-CKD participants, CKD participants had similar carotid maximum wall thickness (2.0 (interquartile range 1.2 to 3.1) mm versus 1.6 (interquartile range 1.1 to 2.9) mm, *p* = 0.13) and similar prevalence of any plaque (62.8% versus 55.6%, *p* = 0.12) NC+ plaque (29.0% versus 30.1%, *p* = 0.80) and Ca + plaque (45.0% versus 44.9%, *p* = 0.99).

**Fig. 2:**
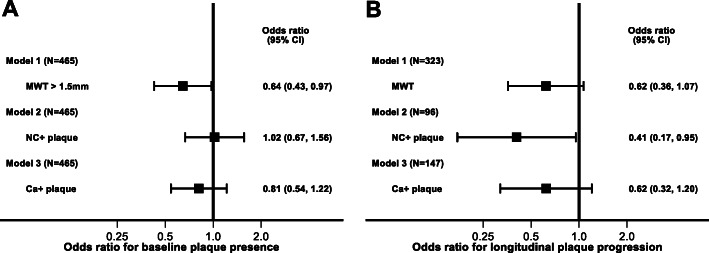
Baseline and longitudinal associations of CKD status with carotid plaque occurrence and plaque morphology. Panel **a***.* Results of separate logistic regression models relating CKD status with MWT > 1.5 mm, NC+ plaque or Ca + plaque adjusted for age, gender, race, CVD history, statin use, and Framingham risk score group in all 465 participants. Panel **b**: Results of separate ordinal logistic regression models relating CKD status with categories of <− 20, − 20% to 20, > 20% longitudinal change in the given plaque type adjusted for age, gender, race, CVD history, statin use, Framingham risk score group and BP intervention arm, stratified by MRI site. Model 1 included all 323 participants with data, model 2 included 96 participants with NC+ plaque at baseline and model 3 included 147 participants with Ca + plaque at baseline. All models included only participants with non-missing quantitative follow-up MRI

In multivariable logistic regression models, adjusted for age, gender, race, baseline CVD and Framingham risk group, compared to the non-CKD subgroup, CKD subgroup had lower odds of baseline prevalence of maximum wall thickness > 1.5 mm (OR 0.64, 95% 0.43 to 0.97, *p* = 0.037) but was not associated with NC+ plaque (OR 1.02, 95% CI 0.67 to 1.56, *p* = 0.92) or Ca + plaque (OR 0.81, 95% CI 0.54 to 1.22, *p* = 0.34) (Fig. 2, Panel a).

### Follow-up MRI

Of the 465 participants with valid baseline MRI (Supplemental Fig. 2), 12 participants died before the follow-up MRI, 8 were lost to follow-up/ inactive in the parent study and 70 did not undergo the follow-up MRI for various reasons (mostly for logistical reasons as the parent study ended earlier than planned). Of the 345 (74.1%) that had follow-up MRI obtained, 14 did not meet quality control standards, 8 had MRIs with qualitative data for plaque composition only and 323 had MRIs with both qualitative data for plaque composition and quantitative data for maximum wall thickness.

### Longitudinal associations of CKD with maximum wall thickness, carotid plaque and plaque morphology

The descriptive associations of CKD status with longitudinal associations of maximum wall thickness, necrotic core (NC) volume and calcification (Ca) volume are presented in Table 2. Of note, only four participants developed new NC+ plaque in the follow-up MRI in those without NC+ plaque at baseline, and none developed new Ca + plaque in the follow-up MRI in those without baseline Ca + plaque.

**Table 2:** Longitudinal changes^a^ in MRI characteristics by CKD subgroups

	Non-CKD Subgroup	CKD Subgroup	*P*-value
Maximum wall thickness change^b^	*N* = 192	*N* = 131	
Baseline maximum wall thickness (mm)	1.9 (1.2, 3.1)	1.7 (1.2, 2.9)	0.38
Follow-up maximum wall thickness (mm)	2.1 (1.2, 3.3)	1.7 (1.2, 3.0)	0.16
Change in maximum wall thickness (mm)	0.03 (−0.14, 0.28)	0.00 (− 0.25, 0.15)	0.085
NC+ plaque present at baseline^c^	*N* = 57	*N* = 39	
Baseline NC+ plaque volume (mm^3^)	17 (10, 30)	15 (10, 34)	0.80
Follow-up NC+ plaque volume (mm^3^)	18 (10, 36)	14 (5, 25)	0.09
Change in NC+ plaque volume (mm^3^)	1.0 (−3.8, 6.5)	−2.0 (−8.7, 1.3)	0.043
Ca + plaque present at baseline ^d^	*N* = 85	*N* = 62	
Baseline Ca + plaque volume (mm^3^)	10 (5, 22)	12 (6, 20)	0.89
Follow-up Ca + plaque volume (mm^3^)	14 (6, 29)	11 (6, 19)	0.17
Change in Ca + plaque volume (mm^3^)	2.4 (−1.5, 6.3)	1.7 (−1.5, 4.2)	0.28

^a^Results are presented as median with interquartile range, *p*-values are by Wilcoxon rank sum test

^b^In those with quantitative maximum wall thickness data at baseline and follow-up (*N =* 323)

^c^In those with NC+ plaque at baseline (*N* = 96)

^d^In those with Ca^+^ plaque at baseline (*N* = 147)

In participants with valid baseline and follow-up maximum wall thickness data (*N* = 323), compared to non-CKD, the CKD was not associated with progression of maximum wall thickness (OR 0.62, 95% 0.36 to 1.07, *p* = 0.08) (Fig. 2, Panel b). In participants with NC+ plaque at baseline and with a follow-up MRI (*N* = 96), CKD subgroup had a lower odds of progression of NC+ plaque (OR 0.41, 95% CI 0.17 to 0.95, *p* = 0.038) (Fig. 2, Panel b). However, CKD was not associated with calcified plaque progression (OR 0.62, 95% CI 0.32 to 1.20, *p* = 0.16) (Fig. 2, Panel b). In spline regression models relating baseline eGFR as a continuous variable with above outcomes, the results were consistent with the main models. (Supplemental Figs. 3 and 4).

### Associations of carotid plaque and plaque morphology and CKD status with CVD outcome

**Fig. 3:**
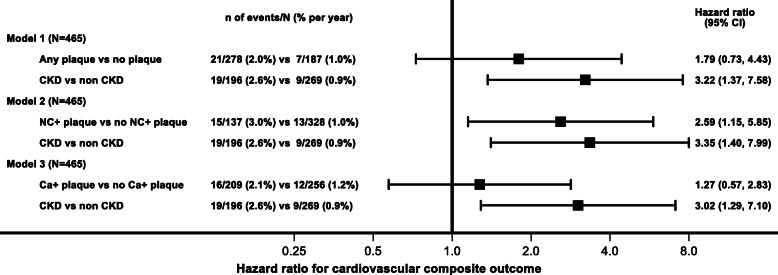
Forest plots of associations of carotid plaque and plaque morphology and CKD status with CVD outcome. Results of separate Cox regression models relating any, NC+ or Ca + plaque and CKD status with the cardiovascular composite outcome. Models were adjusted for age, gender, race, CVD history, statin use, Framingham risk score group and BP intervention arm and stratified by MRI site

The primary CVD composite event rate was 1.59% per year (28 events over 1764 years of follow-up) in the entire cohort. The event rates in persons with and without CKD, any plaque, NC+ plaque and Ca + plaque are summarized in Fig. 3. In separate multivariable Cox regression models adjusted for age, gender, race, CVD history, Framingham risk score group, statin and BP intervention arm and stratified by MRI site, only NC+ plaque (HR 2.59, 95% CI 1.15 to 5.85) but not any plaque (HR 1.79, 95% CI 0.73 to 4.43) or Ca + plaque (HR 1.27, 95% CI 0.57 to 2.83) were associated with increased hazard of the CV composite event.

However, in the above multivariable Cox models, irrespective of adjustment for the presence of any plaque or NC+ plaque or Ca + plaque, CKD was associated with increased risk of CVD composite (Fig. 3).

In sensitivity analyses, associations of plaque presence, plaque morphology and CKD with CV composite without heart failure were similar to the above models (Supplemental Fig. 5).

### Associations of BP intervention with maximum wall thickness changes, NC+ plaque progression and primary CVD composite event

Compared to participants in the standard SBP arm (*N* = 218), those in the intensive SBP arm (*N* = 247) at baseline had similar maximum wall thickness (2.2 ± 0.1 versus 2.4 ± 0.1, *p* = 0.09) and NC+ plaques (30.7% versus 28.3%, *p* = 0.57). In longitudinal models adjusted for age, sex, race, baseline CVD, CKD, Framingham risk score group and statin use, there were non-significantly lower odds ratios of maximum wall thickness progression (OR 0.82, 95% CI 0.48 to 1.38) and NC+ progression (OR 0.61, 95% CI 0.28 to 1.35) in those on intensive SBP arm compared to the standard SBP arm. There were 16 primary CVD composite over 813 person-years of follow-up in the standard arm and 12 primary CVD composite over 951 person-years of follow-up in the intensive SBP arm with a corresponding hazard ratio of 0.75 (95% CI 0.34 to 1.64) in a Cox regression model adjusted for above covariates plus the presence of baseline NC+ plaque.

## Discussion

The results of the current study, one of the largest carotid MRI studies, have several implications. First, presence of necrotic core was more predictive of a CV event than presence plaques defined by maximum wall thickness or calcification. Second, both CKD and NC+ plaque were predictors of CV events, but CKD was not associated with greater prevalence of NC+ plaque at baseline or subsequent faster progression of NC+ plaque.

While vascular calcification as measured by computed tomography (CT) imaging is considered to a strong predictor of CV events in both non-CKD and CKD populations [27, 28], the results of the current study raise the possibility that it might not be the calcification per se but the underlying necrotic core of the plaques that indicates higher risk of CV events. The observation that NC+ plaque rather than Ca + plaque predicted CV events is consistent with the carotid MRI substudy [29] of Atherothrombosis Intervention in Metabolic Syndrome with Low HDL/High Triglycerides: Impact on Global Health Outcomes (AIM-HIGH). In that study of 212 participants at high risk of CV events, NC+ plaques were noted in 52% and Ca + plaques were noted in 48% at baseline and only high lipid content but not high calcification content was predictive of CV events.

Nearly 45 years ago, Lindner et al. proposed the paradigm of accelerated atherosclerosis in uremia [5]. In cross-sectional studies, compared to healthy controls, persons with CKD [8–10], persons with advanced CKD not yet on dialysis (mean serum creatinine 6.4 ± 0.5 mg/dl) [6], and persons on chronic maintenance hemodialysis [6, 7] had higher common carotid artery intima-media thickness. In the current study, we noted that both NC+ carotid plaques and CKD predicted CV events independent of each other.

Based on the accelerated atherosclerosis in CKD hypothesis, we expected to find a higher prevalence of NC+ plaques at baseline and a faster progression of NC+ plaques in persons with CKD compared to those without CKD. Surprisingly, in the current study, we did not observe such associations. One possibility is that non-CKD participants enrolled in the parent SPRINT study were recruited to the study because they were at higher CV risk and hence, the associations of CKD with atherosclerosis might be masked by comparison of a high CV risk non-CKD subgroup. However, as noted above, compared to the non-CKD subgroup, CKD subgroup in the current study had a higher risk of CV events. Hence, selection bias is unlikely to be the explanation for the lack of increased atherosclerotic burden and progression in CKD participants in the current study.

Indeed, these findings are consistent with earlier studies [11–13]. In a longitudinal study of 318 prevalent CKD patients with initial creatinine clearance between 20 and 50 ml/min/1.73 m^2^, plaque area was determined every 6 months using bilateral carotid ultrasonography. Paradoxically, the rate of plaque progression appeared lower in patients with the lowest creatinine clearance [11]. In a meta-analysis of three longitudinal coronary intravascular ultrasound studies of 989 participants [12], there was no difference in progression rates of total atheroma volume and percent atheroma volume in patients with GFRs lower and higher than 60 ml/min/1.73 m^2^. In another longitudinal study of carotid and femoral ultrasounds in 1553 participants with stage 3 to 5 CKD [13], baseline CKD stage was associated with neither the baseline prevalence of atherosclerotic plaque nor the progression of atherosclerotic plaques as evaluated by repeat ultrasound evaluation at 24 months. However, the authors noted that those with progression of kidney disease also had progression of plaques which does not clarify whether those at risk of atherosclerosis progression were also at risk of progression of CKD, or whether progression of CKD lead to progression of atherosclerosis. Taken together, the results of the current study and the previous observations raise the possibility that the increased CV risk observed in the CKD population might be mediated largely by mechanisms other than progression of atherosclerosis such as arterial media calcification or stiffening. It should be noted that carotid MRI could measure plaque calcification [14, 15] but not arterial medial calcification [30].

There are limitations to the current study. First, most of the CKD participants in SPRINT had mild to moderate CKD but not stage 4 or 5 CKD. Nonetheless, the moderate CKD in SPRINT participants was a strong risk factor for CV events. Second, the duration of follow-up in the current study might not have been long enough to capture progression of atherosclerosis. Third, even though this is one of the largest carotid MRI studies, there were only 28 CV events, which limits elaborate adjustment for many covariates as well as analyses by the components of the CV composite. Fourth, compared to the parent study CKD cohort, CKD participants in the current study were more likely to be women, less likely to be of black race, smoker and had lower prevalence of baseline cardiovascular disease. Thus, the current study CKD sub-group might be at lower risk of atherosclerosis progression compared to the parent study CKD sub-group. However, the comparison in the current study is with the non-CKD subgroup and the CKD sub-group was at higher risk of CV events compared to the non-CKD subgroup in this sub-group. Furthermore, diabetes was an exclusion criterion in SPRINT. Thus, this study does not preclude associations of CKD with faster atherosclerosis progression in diabetes. Fifth, we adjusted only for baseline statin use. It is conceivable the duration of statin therapy might impact on atherosclerosis progression. Finally, a larger sample size might have provided more definitive evidence of the BP intervention on progression of NC+ plaque. Even though the current study was only about 5% of the total SPRINT cohort, the point estimate for hazard ratio of the CV outcome for the BP intervention was 0.75 which was similar to what was reported for the entire SPRINT cohort [24]. However, because of the smaller sample size of the current study the confidence intervals were wider.

## Conclusion

In summary, both CKD and carotid NC+ plaques were associated with CV events independent of each other. In the current study, we did not find an association of baseline CKD status with greater burden or progression of atherosclerosis. Thus, the increased CV burden in CKD might be mediated by mechanisms other than atherosclerosis.

## Supplementary Information

**Additional file 1.** Supplemental text

**Additional file 2: Supplemental Table 1.** Comparison of baseline characteristics and clinical outcomes of SPRINT-FAST and SPRINT CKD subgroups

**Additional file 3: Supplemental Figure 1.** Histograms of plaque component changes distributions

**Additional file 4: Supplemental Figure 2.** Inclusion Flowchart

**Additional file 5: Supplemental Figure 3.** Splines relating baseline MDRD eGFR and baseline plaque presence

**Additional file 6: Supplemental Figure 4.** Splines relating baseline MDRD eGFR and longitudinal plaque progression

**Additional file 7: Supplemental Figure 5.** Forest plots of associations of carotid plaque and plaque morphology and CKD status with CVD outcome excluding heart failure

## Data Availability

The datasets generated and/or analyzed during the current study are not yet publicly available, but will be available in the future.

## Abbreviations

CKD: Chronic Kidney Disease
SPRINT: Systolic Blood Pressure Intervention Trial
MRI: Magnetic Resonance Imaging
NC +: Necrotic-Core
MWT: Maximum Wall Thickness
CV: Cardiovascular
AHA: American Heart Association
SBP: Systolic Blood Pressure
SPRINT-FAST: Systolic Blood Pressure Intervention Trial- Factors affecting Atherosclerosis Study
MDRD: Modification of Diet in Renal Diseases
T1w: T1-weighted
T2w: T2-weighted
PDw: Proton-Density-weighted
TOF: Time-Of-Flight
ANOVA: Analysis of variance
CT: Computed Tomography
AIM-HIGH: Atherothrombosis Intervention in Metabolic Syndrome with Low HDL/High Triglycerides Impact on Global Health Outcomes
