# Supplementary material for: Chronic kidney disease, atherosclerotic plaque characteristics on carotid magnetic resonance imaging, and cardiovascular outcomes
Source: BMC Nephrol. 2021 Feb 24;22:69. doi: 10.1186/s12882-021-02260-x (PMC7905597; doi:10.1186/s12882-021-02260-x)
Supplement: Supplementary file 1 — Additional file 1. Supplemental text [file 12882_2021_2260_MOESM1_ESM.docx]

**Supplemental Text:**

Carotid MRI protocol standardization: The MRI protocol was developed centrally by SPRINT-FAST MRI Reading Center at Vascular Imaging Lab, University of Washington, Seattle and transferred in an electronic format for direct implementation to each of the 8 SPRINT-FAST MRI performing sites. Detailed instructions were provided on imaging sequences, artifacts, and pitfalls during site training. Site qualification was based on phantom and volunteer scans which were evaluated by the core lab for protocol adherence. In addition, each participant scan during the study was transferred to the core lab for image quality assessment and timely feedback on potential protocol deviations. Image quality ^28^ was assessed using a 4-point scale (4 = best) based on the overall signal-to-noise ratio and any artifacts that may interfere with image review. Scans that did not meet image quality requirements (image quality <2) were repeated. Repeat scans that still did not meet image quality requirements were excluded from the study.
