# Supplementary material for: Chronic kidney disease, atherosclerotic plaque characteristics on carotid magnetic resonance imaging, and cardiovascular outcomes
Source: BMC Nephrol. 2021 Feb 24;22:69. doi: 10.1186/s12882-021-02260-x (PMC7905597; doi:10.1186/s12882-021-02260-x)
Supplement: Supplementary file 2 — Additional file 2: Supplemental Table 1. Comparison of baseline characteristics and clinical outcomes of SPRINT-FAST and SPRINT CKD subgroups [file 12882_2021_2260_MOESM2_ESM.docx]

**Supplemental Table 1. Comparison of baseline characteristics and clinical outcomes of SPRINT-FAST and SPRINT CKD subgroups:**

|  | SPRINT FAST  CKD* Subgroup  (N=196) | SPRINT  CKD* Subgroup  (N=2,645) |
| --- | --- | --- |
| Estimated GFR^$^, (ml/min/1.73 m2) | 49 ± 8 | 48 ± 10 |
| Age, (year) | 73 ± 8 | 72 ± 9 |
| Age ≥ 75, (%) | 42 | 44 |
| Female sex, (%) | 50 | 40 |
| Black race, (%) | 16 | 24 |
| Statin use, (%) | 46 | 51 |
| Past or current smoker, (%) | 44 | 55 |
| SPRINT intensive SBP arm, (%) | 51 | 50 |
| Systolic blood pressure, (mmHg) | 141 ± 14 | 139 ± 16 |
| Diastolic blood pressure, (mmHg) | 76 ± 11 | 75 ± 12 |
| Pulse pressure, (mmHg) | 65 ± 13 | 64 ± 16 |
| CVD subgroup, (%) | 13^#^ | 24^#^ |
| Urine albumin creatinine ratio, (mg/g) | 11 (6, 41) | 13 (6, 43) |
| Fasting LDL cholesterol (mg/dl) | 110 ± 32 | 107 ± 34 |
| Fasting HDL cholesterol (mg/dl) | 54 ± 15 | 53 ± 15 |
| SPRINT Primary event rate, per 100 person years | 2.59 | 3.20 |
| SPRINT mortality, per 100 person years | 1.44 | 2.37 |

Results are presented as proportions (for binary variables) or as mean ± SD (for continuous variables other than) or as median with interquartile range (for ACR).

^$^ Estimated by 4-variable MDRD equation

*eGFR < 60

**eGFR < 60, age ≥ 50

^#^ CVD subgroup was defined as one or more of MI, ACS, coronary revascularization, carotid revascularization, PAD with revascularization, >50% stenosis of coronary/carotid/lower extremity artery; or AAA ≥5 mm

^&^History of CHF, MI, CHD or stroke
