# Supplementary material for: Chronic kidney disease, atherosclerotic plaque characteristics on carotid magnetic resonance imaging, and cardiovascular outcomes
Source: BMC Nephrol. 2021 Feb 24;22:69. doi: 10.1186/s12882-021-02260-x (PMC7905597; doi:10.1186/s12882-021-02260-x)
Supplement: Supplementary file 3 — Additional file 3: Supplemental Figure 1. Histograms of plaque component changes distributions [file 12882_2021_2260_MOESM3_ESM.docx]

**Supplemental Figure 1** Histograms of plaque component changes distributions
