# Supplementary material for: Chronic kidney disease, atherosclerotic plaque characteristics on carotid magnetic resonance imaging, and cardiovascular outcomes
Source: BMC Nephrol. 2021 Feb 24;22:69. doi: 10.1186/s12882-021-02260-x (PMC7905597; doi:10.1186/s12882-021-02260-x)
Supplement: Supplementary file 4 — Additional file 4: Supplemental Figure 2. Inclusion Flowchart [file 12882_2021_2260_MOESM4_ESM.docx]

**Supplemental Figure 2: Inclusion Flowchart**

Did not attend baseline clinic visit (n=39)

Did not attend baseline MRI visit (n=55)

Baseline MRI required repeat (n=53)

Baseline MRI required 2nd repeat (n=3)

Baseline MRI did not meet QC (n=36)

SPRINT Trial Participants

Enrolled in SPRINT FAST (n=595)

Completed baseline clinic visit (n=556)

Completed baseline MRI visit (n=501)

Baseline MRI met QC and included in analysis (n=465)

Baseline MRI has quantitative data for analysis(n=435)

MRI qualitative only (n=30)

Follow-up MRI with quantitative data (n=323)

Follow-up MRI qualitative only (n=8)

Follow-up MRI did not meet QC (n=14)

Participant deceased (n=12)

Participants lost to follow-up/inactive in SPRINT (n=8)

Logistical reasons* or lack of participant interest(n=70)

*As SPRINT was terminated earlier than planned, follow-up MRIs could not be obtained in this SPRINT ancillary study
