# Supplementary material for: Chronic kidney disease, atherosclerotic plaque characteristics on carotid magnetic resonance imaging, and cardiovascular outcomes
Source: BMC Nephrol. 2021 Feb 24;22:69. doi: 10.1186/s12882-021-02260-x (PMC7905597; doi:10.1186/s12882-021-02260-x)
Supplement: Supplementary file 5 — Additional file 5: Supplemental Figure 3. Splines relating baseline MDRD eGFR and baseline plaque presence [file 12882_2021_2260_MOESM5_ESM.docx]

**Supplemental Figure 3** Splines relating baseline eGFR and baseline plaque presence
