# Supplementary material for: Chronic kidney disease, atherosclerotic plaque characteristics on carotid magnetic resonance imaging, and cardiovascular outcomes
Source: BMC Nephrol. 2021 Feb 24;22:69. doi: 10.1186/s12882-021-02260-x (PMC7905597; doi:10.1186/s12882-021-02260-x)
Supplement: Supplementary file 6 — Additional file 6: Supplemental Figure 4. Splines relating baseline MDRD eGFR and longitudinal plaque progression [file 12882_2021_2260_MOESM6_ESM.docx]

**Supplemental Figure 4** Splines relating baseline eGFR and longitudinal plaque progression
