# Supplementary material for: Chronic kidney disease, atherosclerotic plaque characteristics on carotid magnetic resonance imaging, and cardiovascular outcomes
Source: BMC Nephrol. 2021 Feb 24;22:69. doi: 10.1186/s12882-021-02260-x (PMC7905597; doi:10.1186/s12882-021-02260-x)

**Supplemental Figure 5** : Forest plots of associations of carotid plaque and plaque morphology and CKD status with CVD outcome excluding heart failure.
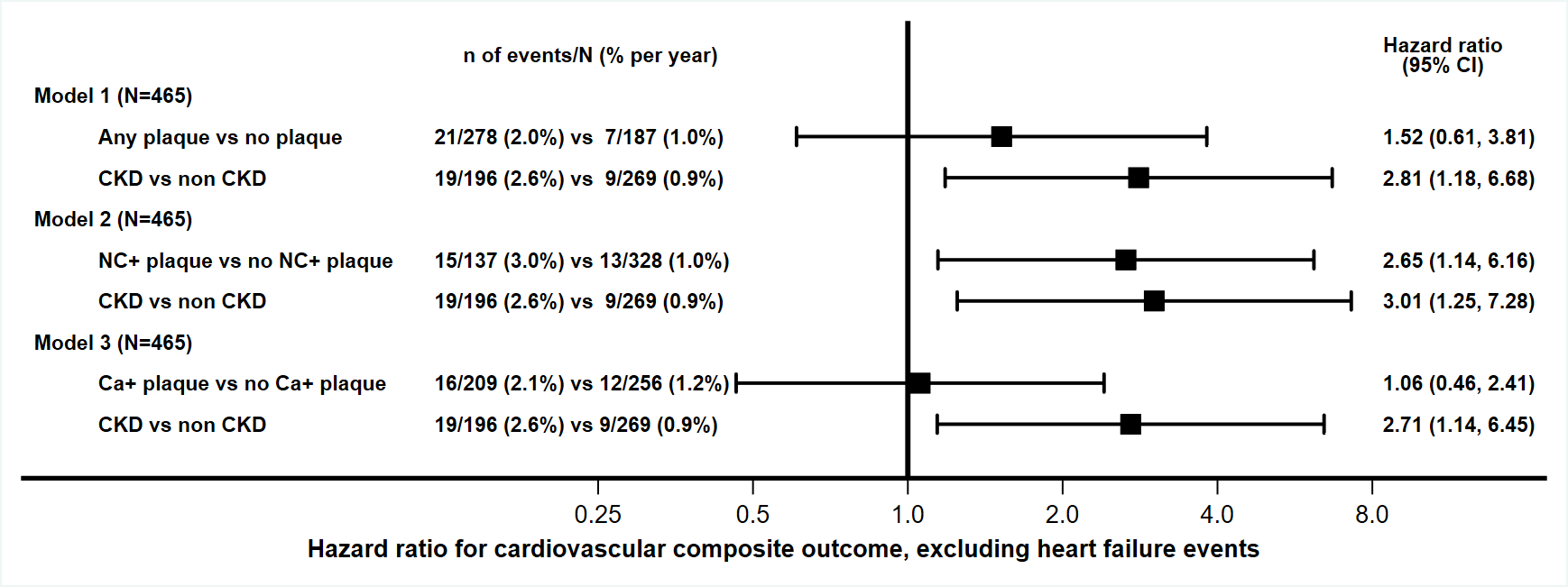

Supplement: Supplementary file 7 — Additional file 7: Supplemental Figure 5. Forest plots of associations of carotid plaque and plaque morphology and CKD status with CVD outcome excluding heart failure [file 12882_2021_2260_MOESM7_ESM.docx]
